# Early Immunologic Events at the Tick-Host Interface

**DOI:** 10.1371/journal.pone.0047301

**Published:** 2012-10-15

**Authors:** Dar M. Heinze, J. Russ Carmical, Judith F. Aronson, Saravanan Thangamani

**Affiliations:** 1 Department of Pathology, University of Texas Medical Branch, Galveston, Texas, United States of America; 2 Institute for Human Infections and Immunity, University of Texas Medical Branch, Galveston, Texas, United States of America; 3 Department of Biochemistry and Molecular Biology, University of Texas Medical Branch, Galveston, Texas, United States of America; Metabiota, United States of America

## Abstract

*Ixodes* species ticks are competent vectors of tick-borne viruses including tick-borne encephalitis and Powassan encephalitis. Tick saliva has been shown to facilitate and enhance viral infection. This likely occurs by saliva-mediated modulation of host responses into patterns favorable for viral infection and dissemination. Because of the rapid kinetics of tick-borne viral transmission, this modulation must occur as early as tick attachment and initiation of feeding. In this study, cutaneous bite-site lesions were analyzed using Affymetrix mouse genome 430A 2.0 arrays and histopathology at 1, 3, 6, and 12 hours after uninfected *Ixodes scapularis* nymphal tick attachment. At 1 and 3 hrs after attachment, the gene expression profile is markedly different than at later time points. Upregulated gene ontology term clusters enriched at 1 and 3 hrs were related to post-translational modification. At 6 and 12 hrs, cytoskeletal rearrangements, DNA replication/cell division, inflammation, and chemotaxis were prominent clusters. At 6 and 12 hrs, extracellular matrix, signaling, and DNA binding clusters were downregulated. Histopathological analysis shows minimal inflammation at 1 and 3 hrs but an appreciable neutrophil infiltrate at 6 and 12 hrs. In addition, putative hyperemia, localized necrosis, and increased ECM deposition were identified. Putting the gene expression and histopathology analysis together suggests early tick feeding is characterized by modulation of host responses in resident cells that merges into a nascent, neutrophil-driven immune response by 12 hrs post-attachment.

## Introduction

Emerging and re-emerging diseases transmitted by blood feeding arthropods are significant global public health problems. Ticks transmit the greatest variety of pathogenic spirochetes, rickettsiae and viruses of any blood feeding arthropod [1]. Infectious agents transmitted by ticks are delivered to the vertebrate host together with saliva at the bite site. Tick salivary glands produce a complex repertoire of bioactive molecules that creates an immunologically privileged microenvironment facilitating blood feeding and pathogen transmission [2]. Ticks remain attached to their hosts for a few hours in the case of soft ticks or several days in the case of hard ticks. To successfully complete their feeding, ticks have evolved strategies to circumvent innate immune responses when feeding on naïve hosts and both innate and adaptive immune responses when feeding on tick-experienced animals. Skin is the interface between the tick and the host. Skin acts as a physical barrier and also contains an array of resident immune cells such as eosinophils, mast cells, dendritic cells, macrophages and keratinocytes. Tick feeding and salivary gland molecules skew host immune response away from T_H_1 and toward a T_H_2 profile [3]. Salivary gland proteins and extracts have been shown to inhibit immune cells at the bite site and dendritic cell maturation and migration [4].

During tick feeding and attachment, significant morphological changes occur in salivary glands [5]. Salivation is not a continuous process during blood feeding [6] and the repertoire of salivary proteins changes during the course of feeding [7], [8]. These temporal patterns allow the saliva proteins of the tick to “prime” the feeding site to different degrees prior to introduction of infectious agents. Not all tick-borne pathogens are transmitted at the same time during the feeding process. Tick-borne encephalitis was observed to be transmitted within the first few hours of attachment/feeding, while *Borrelia burgdorferi* was observed to be transmitted between 24 and 48 hours post tick attachment/feeding [9], [10], [11]. We believe that the temporal expression of immunomodulatory tick salivary proteins secreted into the bite site depends on the pathogen it transmits. In this study, we sought to characterize tick-induced changes in cutaneous gene expression at the earliest stages of attachment/feeding by *I. scapularis* nymphs using Mouse Genome microarrays. This will allow us to understand immunomodulation at the tick-host interface induced by tick saliva that facilitates tick-borne virus transmission.

## Methods

### Ethics Statement

All experiments were conducted in an arthropod containment level 2 (ACL-2) facility in accordance with an animal use protocol approved by the University of Texas Medical Branch (UTMB) Institutional Animal Care and Use Committee (IACUC).

### Animals

BALB/c mice used in this study were obtained from The Jackson Laboratory (Bar Harbor, ME). Mice were cared for in accordance with guidelines of the Committee on Care and Use of Laboratory Animals (Institute of Laboratory Animal Resources National Research Council, Washington, DC).

### Tick Colony Maintenance and Infestations

*Ixodes scapularis* ticks were maintained as described in [12] and [13]. Eight-week old BALB/c mice were anesthetized with a 150 υL intraperitoneal injection containing 10 mg/mL ketamine (Fort Dodge Animal Health, Fort Dodge, IA) and 1 mg/mL xylazine (Phoenix Pharmaceutical, St. Joseph, MO) in PBS (Gibco, Life Technologies, Carlsbad, CA). Eight *Ixodes scapularis* nymphs were placed on the ears of each mouse and observed until attachment. Mice were euthanized by an additional injection of 200 υL anesthetic followed by cervical dislocation at 1, 3, 6, and 12 hours after tick attachment. Skin biopsies with attached ticks were harvested using 4 mm biopsy punches (Premier Products Co., Plymouth Meeting, PA). Control samples were harvested in the same way from tick-free mice. For the micro-array experiment, 3 mice were used at each time point; for the real-time PCR validation experiment, 4 mice were used at each time point. Skin biopsies were preserved in RNALater (Ambion, Life Technologies, Carlsbad, CA) or 10% neutral buffered formalin. The Institutional Animal Care and Use Committee of the University of Texas Medical Branch approved all animal protocols.

### RNA Extraction

Ticks were removed from all skin biopsies before RNA extraction. Tissue samples were homogenized individually in 1 mL Trizol (Life Technologies, Carlsbad, CA) using an Ultra-Turrax T8 (Ika, Wilmington, NC) tissue disperser. After 5 min incubation, 200υL chloroform (Fisher Scientific, Waltham, MA) was added and the samples incubated for 3 min. Samples were centrifuged at 12,000×*g*, 4°C for 15 min, and the aqueous phase was retained. One volume 70% ethanol (Acros Organics) was added and the samples applied to RNeasy micro kit (Qiagen) columns. The RNeasy protocol was then followed, including the in-column DNase digestion step. After extraction, RNA was quantitated spectrophotometrically using a NanoDrop ND-1000 (NanoDrop Technologies, DE). All samples were required to read greater than 1.8 on both A_260_/A_280_ and A_260_/A_230_ ratios. For subsequent microarray analysis, quality of the purified RNA was assessed by visualization of 18 S and 28 S RNA bands using an Agilent BioAnalyzer 2100 (Agilent Technologies, CA). Resulting electropherograms were used in the calculation of the 28S/18S ratio and the RNA Integrity Number, which was greater than 6.8 in all samples [14]. For subsequent real-time PCR analysis, RNA integrity was determined by denaturing (formaldehyde) agarose gel electrophoresis followed by staining with Sybr Gold stain (Invitrogen). Visualization of clear ribosomal bands indicated minimal degradation. Eluted RNA samples were aliquoted and stored at −80°C until use.

### Affymetrix GeneChip Gene Expression Analysis

Total RNA (500 ng) was converted to cRNA for microarray analysis using the Ambion MessageAmp™ Premier RNA Amplification Kit (Life Technologies Corporation, CA) according to manufacturer's instructions. Total fragmented cRNA (10υg) was hybridized to the Affymetrix GeneChip Mouse Genome 430A 2.0 array according to the manufacturer’s (Affymetrix, CA) conditions. The chips were washed and stained in a GeneChip Fluidics Station 450 and fluorescence detected with an Affymetrix-7G Gene Array scanner using the Affymetrix GeneChip Command Console software (AGCC1.1). Raw data can be accessed through Gene Expression Omnibus record GSE39100.

### Gene Expression Data Analysis

Gene expression changes in comparison to tick-free mice were identified using Partek Genomics Suite (Partek, MO) following the default gene expression workflow. The resulting values were then filtered for p-values ≤0.05 and a fold change ≤ −1.5 or ≥ +1.5. Lists of up and downregulated genes at each time point were individually submitted to the Database for Annotation, Visualization, and Integrated Discovery (DAVID) [15], [16] website using the Mouse Genome 430A 2.0 array as a background list. The functional annotation clustering tool was used to cluster gene ontology terms with shared genes into groups to allow an easier functional understanding of the array data. Gene expression data was also entered into ingenuity pathway analysis software (Ingenuity Systems, Redwood City, CA).

### Real-time PCR Validation

A list of 28 genes (Table 1) were chosen for further real-time PCR validation based on significant fold change in the array data at 12 hpi or previously identified genes of interest in host anti-tick responses [13]. Pre-optimized primer pairs for these genes were purchased from SABiosciences (Qiagen); primer sequences are property of Qiagen. Primers were mixed with RT^2^ SYBR green qPCR master mix (Qiagen) and aliquoted into 20 iCycler iQ PCR plates (Bio-Rad) using an epMotion 5075 automated pipetting system (Eppendorf). Plates were sealed and stored at −20°C until use. For each real-time PCR run, the RT^2^ First Strand Kit (Qiagen) was used to convert 1 µg total RNA into cDNA, which was then loaded onto PCR plates using the epMotion 5075 automated pipetting system (Eppendorf). These plates were run on an iCycler iQ5 real-time PCR instrument (Bio-Rad) with the following cycling protocol: 10 min at 95°C; 15 s at 95°C, 1 min 60°C for 40 cycles, and an 80-cycle (+0.5°C/cycle) 55–95°C melt curve. Every run included hypoxanthine guanine phosphoribosyl transferase (Hprt) and heat shock protein 90 alpha (Hsp90ab1) as endogenous control genes, and ‘no template’ and ‘no first strand’ controls. HTqPCR, an R-based program for real-time PCR data analysis [17], was used to analyze data using the delta-delta Ct method for gene expression normalization and measurement, and the linear models in microarray analysis (LIMMA) package for statistical comparisons between infested and tick-free mice, as previously described [13].

**Table 1:** Gene list for real-time PCR validation.

Banf1	Fn1	Il2	Socs1
C1qb	Foxp1	Il4ra	Stat6
Ccl2	IFN-γ	Jak1	Vapb
Ccl7	IL-10	Muc1	Vwf
Ccr5	IL-1β	Myb	Hprt1
Clec4e	IL-3	Saa1	Hsp90ab1
Ctse	IL-4	Sele	No RT
Cxcl5	IL-6	Serpina3n	No temp

### Histological Analysis

Skin biopsies were fixed for a minimum of 48 hours in 10% neutral buffered formalin, treated for 2 hours with decal (Decal Chemical Corp, Tallman, NY), and embedded in paraffin with special attention to orient the tissue sample to allow a cross-section of the skin and a longitudinal section of the tick upon sectioning. Paraffin blocks were carefully sectioned and the slides containing the entry of the hypostome into the epidermis were retained for H&E and Giemsa staining.

## Results and Discussion

### Microarray Analysis of Host Immune Responses to Early Tick Feeding

**Figure 1:**
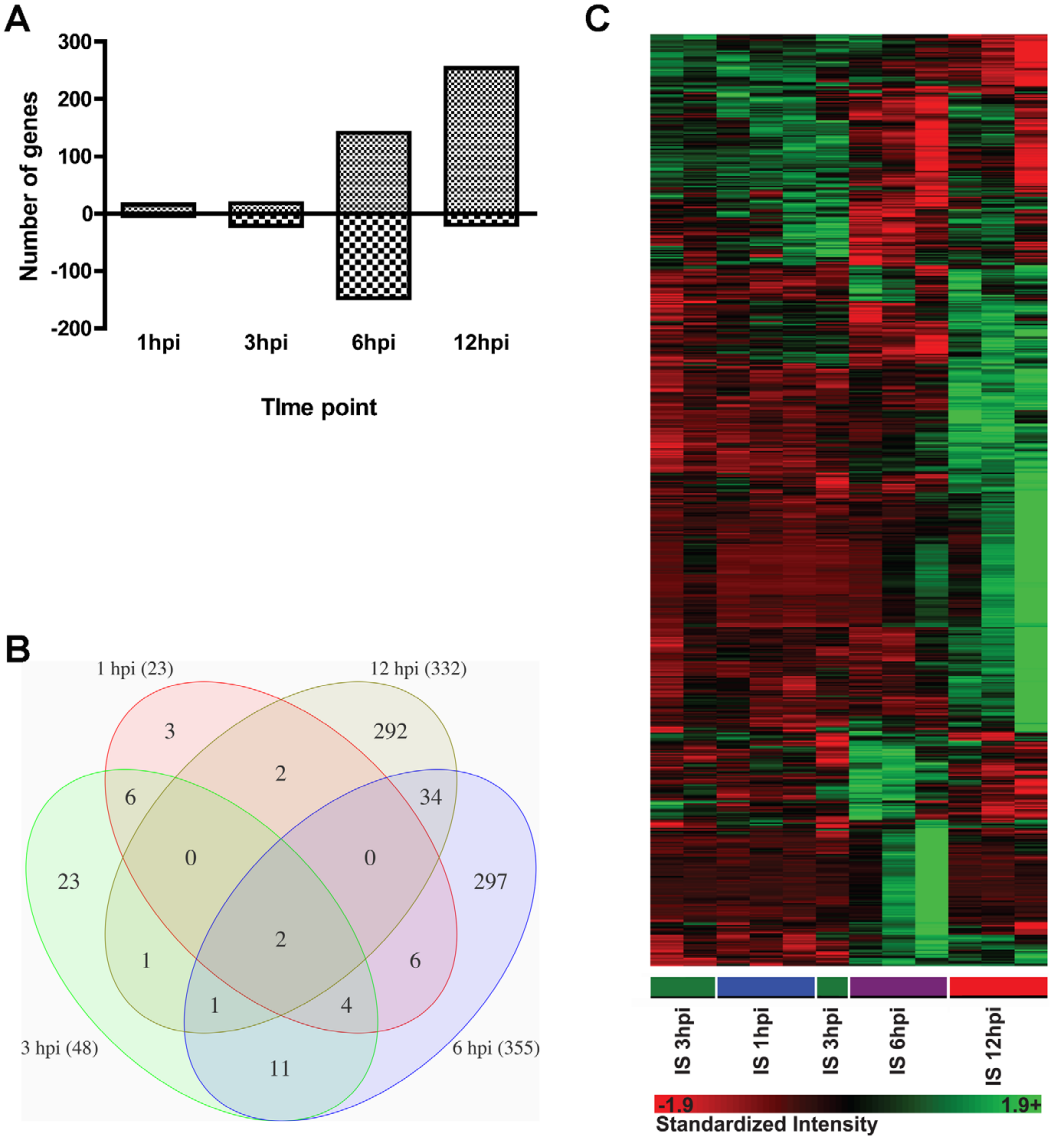
An overview of gene expression profiles from tick bite sites at 1, 3, 6, and 12 hours post-infestation. The immune response at the tick-host interface was investigated at 1, 3, 6 and 12 hours post nymphal tick infestation (hpi) using mouse Affymetrix GeneChip microarrays. A: Number of significantly up and downregulated genes measured at each time point during tick infestations of mice with *I. scapularis* nymphs compared to tick-free mice; B: Venn diagram showing overlap of significantly modulated genes between time points; C: Differential gene expression data was used to generate a heat map using Partek Genomics analysis suite showing temporal changes in gene expression profiles.

The immune response at the tick-host interface was investigated at 1, 3, 6 and 12 hours post nymphal tick infestation (hpi) using microarrays. Significantly modulated genes increased across time (Figure 1a), reflecting the development of host responses as the infestation progressed. A higher percentage of modulated genes were shared with adjacent than distant time points (Figure 1b). The specific gene expression profiles were similar between 1 and 3 hours, but showed appreciable change between 3 and 6 hours, and again between 6 and 12 hours post-infestation (Figure 1c). These results suggest cutaneous responses undergo rapid changes in gene expression profiles in the early stages of tick feeding.

Significantly modulated genes were divided into up and downregulated lists at each time point and submitted to the Database for Annotation, Visualization and Integrated Discovery (DAVID) bioinformatics database. The functional annotation-clustering tool was used to group similar terms together. These clusters were then named according to the gene ontology terms in each cluster (Table 2). At 1 and 3 hrs post-infestation, the only significantly up-regulated cluster was “post-translational modification: ubiquitin, isopeptide;” no significantly downregulated clusters were observed. Changes in gene expression at 1 and 3 hrs post-infestation were related to signaling pathways such as NFκB and cation homeostatsis, suggesting pro-inflammatory pathways are already activated. At 6 hrs post-infestation, clusters related to cytoskeletal elements, keratinocyte migration, cell signaling, transcription, and immune responses were prominent. At 12 hrs post-infestation, cell cycle, cytoskeletal elements, and immune response clusters were observed. Immune response clusters differed between 6 and 12 hrs post-infestation. At 6 hrs, anti-microbial responses, immunoreceptor signaling, and negative regulation of myeloid differentiation were significant, while at 12 hrs, inflammation and chemotaxis were significant. These results suggest a rapid, pro-inflammatory evolution of the early host response beginning soon after attachment that progress from early inflammatory signaling and pre-programmed anti-microbial responses to the infiltration of innate immune cells by 12 hpi.

**Table 2:** Gene ontology clusters from DAVID analysis.

Clusters from upregulated genes	
6 hpi	12 hpi
Cytoskeleton, intermediate filament, keratin filament,non-membrane bound organelle	Cytoskeleton: intermediate filament
Transcription factor, regulation of transcription, DNA binding	Keratin, intermediate filament
Epithelial development, keratinocytes, cytoskeleton	DNA replication
Transcription and RNA metabolism	DNA repair
Protein phosphatase	Cytoskeleton organization: intermediate filament
Cation homeostasis	Epidermis development, hair follicle cycle
Anti-microbial response	Inflammation, chemotaxis
Negative regulation of myeloid cell differentiation	Epithelial development
B-cell, T-cell, and Toll-like receptor signaling	Cell cycle
	Nucleolus, membrane-enclosed lumen
**Clusters from downregulated genes**	
**6 hpi**	**12 hpi**
Secreted: signal peptide, disulfide bond, glycosylation	Skeletal system development
Armadillo repeat-containing proteins	Regulation of transcription, DNA binding
Von Willebrand factor C domain	Internal side of plasma membrane, organelle organization
G protein signaling domain	
Extracellular matrix	
Skeletal system: ossification	
Cell and cell-cell adhesion	
Blood vessel development	
Transmembrane, glycoprotein	
Golgi, cytoplasmic vesicle, clathrin coated vesicle	

### Cytoskeletal Changes

At both 6 at 12 hpi, the most significantly upregulated gene ontology clusters were related to components of the cytoskeleton such as intermediate filaments. A closer look at these genes revealed many keratin intermediate filament transcripts. Keratin intermediate filaments have been shown to protect epithelial tissues from mechanical and non-mechanical stresses, modulate apoptosis, regulate some aspects of skin pigmentation, and control keratinocyte migration during the process of wound healing [18], [19], [20], [21]. Because the initiation of the feeding lesion necessitates significant local damage to epithelial tissues, we believe these ontology terms likely reveal early epithelial attempts to close the wound. Interestingly, Krt6, a gene upregulated at both 6 and 12 hpi has already been shown to be upregulated in keratinocytes at wound margins as early as 6 hrs post injury, and to pair with Krt16/17 during wound healing [21]. The upregulation of all these genes at 12 hpi in our study supports the possibility these gene ontology clusters describe a nascent wound healing response at the tick bite site.

### Transcription Factors and Cell Signaling Pathways

A number of gene ontology clusters were related to signaling pathways and downstream targets such as transcription factors. Upregulated clusters at 6 hpi were transcription/transcription factors and protein phosphatase, while downregulated clusters included G-protein signaling domain. While many transcription factors were modulated, the largest group belonged to members of the activator protein-1 (AP-1) transcription factor family. AP-1 is composed of homo- or hetero- dimers of Fos, Jun, Atf3, and Maff proteins. It can activate an extremely broad range of processes that include oncogenesis, regulation of bone homeostasis, and immunity. In the skin, loss of JunB leads to a systemic lupus erythematosis-like phenotype, while loss of both c-Jun and JunB leads to postnatal death. An inducible deletion of c-Jun and JunB in the skin leads to a psoriasis-like phenotype characterized by joint inflammation, hyperkeratosis, and epidermal infiltrates [22]. Thus AP-1 members play a significant role in regulating epidermal inflammatory responses. Our data suggests AP-1 may be activated through the mitogen-activated protein kinase (MAPK) pathway, based on the upregulation of Mak3k6 and negative regulators of the MAPK pathway such as dual-specificity phosphatases (DUSP). In addition to AP-1, members of the NFκB family such as Nfkbia, Ikbz, and Nfkbiz were also upregulated. NFκB target genes include many pro-inflammatory cytokines and chemokines, including IL-1β, IL-6, and CCL2 that are upregulated in this study.

G-protein signaling domain was the primary downregulated cluster related to signaling. Two groups of G-protein signaling modulators were represented: regulators of G-protein signaling (RGS) and G-protein coupled receptor kinases (GRK). Both of these groups act to dampen GPCR activity. RGS molecules accomplish this by enhancing the intrinsic GTPase activity of activated Ga subunits [23] while GRK proteins phosphorylate the active GPCR, making it a high-affinity target for arrestin binding which blocks G-protein binding and activation [24]. Thus the downregulation of these molecules may potentiate GPCR signaling at the tick bite site.

### Immune Response

A number of clusters in the gene ontology analysis at 6 hpi were related to the immune response. These were cation homeostasis, anti-microbial response, negative regulation of myeloid cell differentiation, and B-cell, T-cell, and Toll-like receptor signaling. Within the cation cluster were transcripts for genes involved with iron, zinc, calcium, and proton transport or regulation. In particular, lactotransferritin, metallothionein 1, and metallothionein 2 have been shown to function in regulating reactive oxygen species production and scavenging [25], [26]. While some of the genes in this cluster are related calcium transport and may function in cell signaling, we suspect that regulating the oxidative status of the tissues near the bite site is the primary function of these genes.

Genes of interest in the anti-microbial cluster were beta-3 defensin (Def3b) and peptidoglycan recognition protein (Pglyrp1). Defensins are small positively charged cysteine-rich peptides with antimicrobial activity; interestingly, epithelial tissues but not neutrophils were the primary sources of mouse beta-defensins [27]. Def3b has wide spectrum anti-microbial activity against bacteria [28], fungi [29], and viruses [30]. Pglyrp1 has been shown to enhance intracellular killing of bacteria in neutrophils [31]. Thus early host responses to tick feeding include upregulation of potent anti-microbial proteins that could impact the transmission of tick-borne pathogens.

Genes within the negative regulation of myeloid cell differentiation and B-cell, T-cell and Toll-like receptor signaling clusters were transcription factors and signaling intermediates mentioned above (see Transcription factors and cell signaling pathways heading).

At 12 hpi, the only gene ontology cluster related to the immune response was inflammation and chemotaxis. The genes in this cluster were cytokines, chemokines, and related molecules. Chemokines are small peptides that are potent activators and chemoattractants for leukocytes, and play an important role at the sites of inflammation. Overall, nymphal tick feeding induced the expression of chemokines specific for neutrophil (Cxcl1 and 5) and monocyte (Ccl2, 6, 7, and 12) recruitment. In addition, Cxcl14 was upregulated, a chemokine specific for dendritic cell precursors [32] but without a defined function in the skin [33]. Increasing evidence suggests that small inflammatory mediators such as leukotrienes, prostaglandins, platelet activating factor, and complement initiate chemotaxis to sites of inflammation. This initial response is amplified by cytokine production that drives chemokine synthesis [34]. Our results support the upregulation of IL-1b, IL-6, and C1qb that may interact with the chemokine profile to maintain and amplify the chemotactic response. While the sequence of events could not be defined in this study, the gene expression profile strongly suggests the recruitment of neutrophils and monocytes to the bite site.

### Ingenuity Pathways Analysis

**Figure 2:**
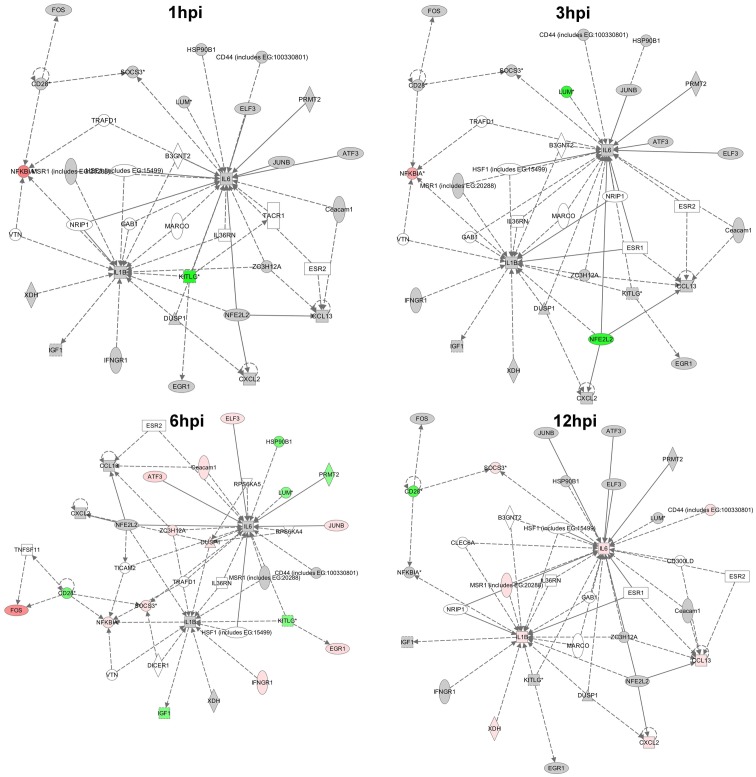
Diagram showing the interactions between genes and the temporal increase in representation of the acute inflammation and immune cell recruitment pathway from the IPA analysis. Gene expression data was entered into ingenuity pathway analysis software to identify potential canonical pathways modulated during tick feeding. Upregulated genes are orange/pink, downregulated genes are green, and unchanged or unsignificant genes are grey.

**Figure 3:**
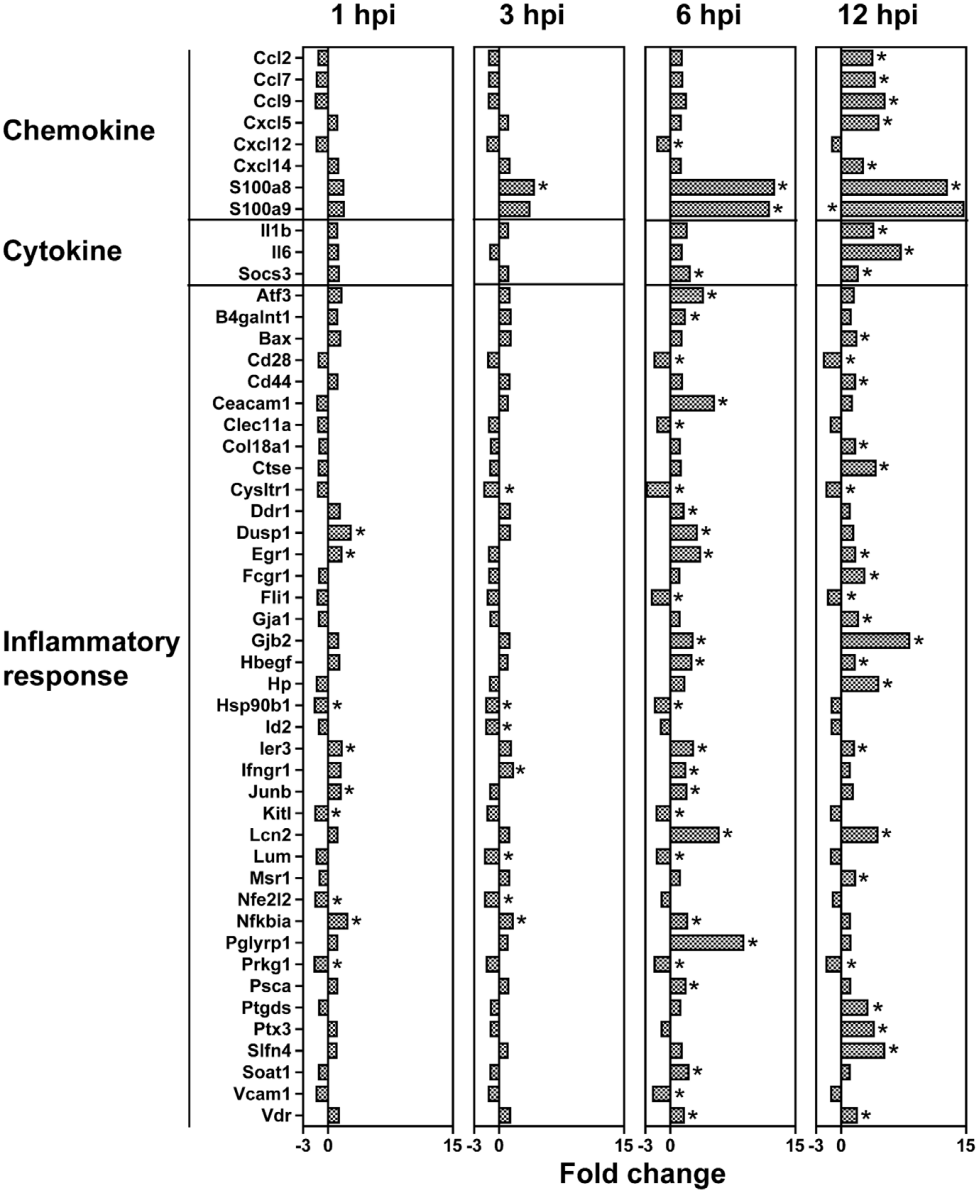
Temporal changes in the immune genes expression during tick feeding. Gene expression data was entered into ingenuity pathway analysis software. All genes significantly modulated at any time point in the inflammatory response pathway from the IPA analysis were plotted showing temporal changes in gene expression. Genes significant at each time point are marked with an asterisk.

All significantly modulated genes were submitted to Ingenuity Pathways Analysis. IPA has some advantages over DAVID in that it takes into account the fold change associated with each modulated transcript when determining biological significance. The most significant pathway in this dataset was related to acute inflammation and immune cell recruitment, supporting the DAVID analysis. The interactions between genes in this pathway were mapped over time (Figure 2), showing temporal increases in gene modulation in this pathway. In addition, all the genes modulated at any time point in the inflammatory response pathway were plotted to show temporal changes in gene expression (Figure 3). We believe this data suggests early tick feeding is characterized by an inflammatory response from the earliest time point that intensifies as feeding continues.

### Validation of Microarray Data

**Figure 4:**
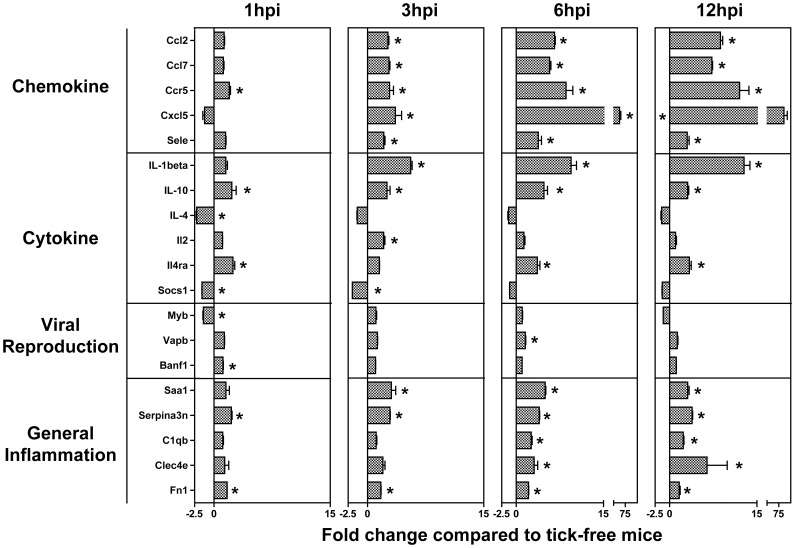
Real time PCR validation of array data. Validation targets were chosen based on significant fold change in the array study at 12 hpi and/or genes previously identified as important in host anti-tick responses [13]. Pre-optimized primers were purchased from Qiagen, and real-time PCR was performed as described in the methods section. All significantly modulated genes at any time point in the validation study are plotted. Significant changes in gene expression at individual time points are marked with an asterisk. Significance was assessed using the delta-delta Ct method for gene expression normalization and measurement, and the linear models in microarray analysis (LIMMA) package for statistical comparisons between infested and tick-free mice, as previously described [13].

Based on the significance of chemotaxis and inflammation in the gene ontology analyses, we chose a list of chemokines, cytokines, inflammatory molecules, and genes in the ontology term “viral reproduction” (GO:0016032) for validation by real-time PCR (Table 1). Specific validation targets were chosen by significant fold change in the array data and/or previously identified genes of interest in host anti-tick responses [13]. All significantly modulated genes at any time point in the validation experiment are shown in figure 4. While exact fold changes did not correlate, trends of up or down regulation were well preserved between the array and validation studies (Table S1). Ctse, Foxp1, IL-6, and Muc1 were modulated in the array but were not validated. Other genes such as Ifng, IL-3, Jak1, Stat6, and Vwf were not modulated in either study. Significant increases in chemokine gene expression begin at 1 hr post-infestation and intensify as time progresses, supporting the importance of chemotaxis seen in the array. Upregulation of IL-1β, IL-10, and IL-4ra along with the general inflammation group supports early inflammatory changes at the bite site. While genes in the viral reproduction group showed some significant modulation, changes between time points were not consistent, casting some doubt on the biological significance of these differences.

### Histopathology

**Figure 5:**
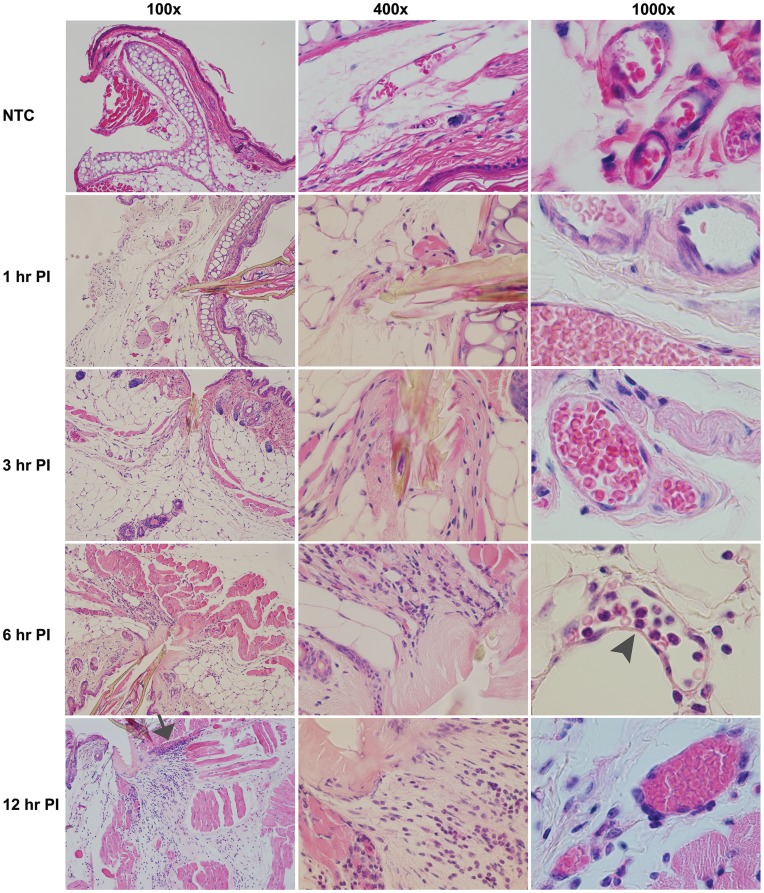
Histopathology of *Ixodes scapularis* nymphal bite sites at 1, 3, 6, and 12 hrs PI. Skin biopsies were fixed in formaldehyde followed by decalcification prior to paraffin embedding. Five micron sections were stained with hematoxylin and eosin, as described in the methods section. The arrowhead marks a marginating neutrophil at 6 hrs PI 1000x, while the arrow marks areas of putative myositis/muscle necrosis at 12 hrs PI 100x.

Concurrently with gene expression measurements, we pursued histopathological analysis of the bite site to see if morphological changes could be correlated with array data. Resident cells such as keratinocytes, fibroblasts, dendritic cells, and mast cells initially sense cutaneous tissue injury or antigenic stimuli. These cells release rapidly synthesized or pre-formed pro-inflammatory and chemotactic molecules including histamine and eicosanoids. These molecules can stimulate cytokine production and endothelial cells to mobilize Weibel-Palade bodies containing pre-formed selectins to the cell surface. While these early events cannot be measured at the transcriptional level, the histopathological analysis clearly shows tissue damage from the insertion of the hypostome and degranulating mast cells (Figure S1) as early as 1 hr post attachment. Minor inflammatory changes consisting of a few inflammatory cells and a small amount of eosinophilic material near the tick hypostome were also observed. By 3 hrs post-infestation, inflammatory cells were readily evident, the eosinophilic material near the hypostome was much more pronounced, and the tissue architecture had the appearance of streaming toward the bite site, even in hypodermal muscle layers. This appearance suggests that ticks may initially insert the hypostome deeply and then retract it, pulling deeper tissues towards the epidermis. These changes intensify at 6 hrs post-infestation, leading to a very intense, neutrophil dominated inflammatory lesion by 12 hrs of tick feeding. Also visible at 12 hrs were potential areas of myositis, muscle necrosis, and increased congestion in blood vessels near the hypostome (Figure 5).

### Early Immunologic Response to Tick Bites

The early appearance of pro-inflammatory changes in transcription and histopathology was unexpected. Previous studies in our laboratory had suggested a minimal early host response [13], supporting many studies that have shown tick salivary components are capable of inhibiting nearly every aspect of cell recruitment. *Ixodes ricinus* saliva contains leukotriene B4 binding proteins that have been shown to reduce neutrophil migration [35], histamine binding proteins have been described from *Rhipicephalus appendiculatus* saliva [36], and numerous tick anti-complement molecules have been described [37], [38], [39]. The release of histamine, eicosanoids, and complement fragments are likely some of the earliest events in the chemotactic cascade. In addition, *I. scapularis* saliva has been shown to downregulate neutrophil beta-2 integrins, reduce phagocytic efficiency, and inhibit intracellular killing of *Borrelia burgdorgeri* [40]. The reduction in intracellular killing may be explained by salivary proteins that block super-oxide formation [41], and detoxify reactive oxygen species [42]. Tick salivary proteins have also been shown to bind human IL-8 [43] and chemokines such as Cxcl8 [44]. These studies show tick saliva can inhibit later events in the chemotactic cascade and also effector functions of neutrophils. Against this backdrop, the present study shows leukocytes such as neutrophils and pro-inflammatory gene transcription was initiated before 3 hours post-infestation. Thus despite the impressive arsenal of inhibitory tick salivary proteins, the host is able to mount a surprisingly timely immune response. Studies in mice with labeled neutrophils (enhanced GFP expression under the control of the lysozyme M promoter) have shown that neutrophils migrate into sites of sterile cutaneous injury as soon as 20 minutes post-injury. Neutrophil numbers then increased rapidly for 2 hrs when a plateau-phase was reached [45]. In a similar model using a larger wound (6 mm) and EGFP- labeled neutrophils, influx was measurable at 4 hrs and did not plateau until 2–3 days post wounding [46]. These studies suggest neutrophil chemotaxis into sites of cutaneous injury was initiated within 20 minutes, but the subsequent kinetics and final concentration of neutrophils may depend on other factors such as the size of the wound. In our study, very few neutrophils were visible at the bite site by 1 hpi. It should be noted that this is 1 hour after apparent tick attachment and hence represents the maximum length of attachment. Even so, it seems likely that the very early phase of neutrophil recruitment to nymphal tick bite sites is slower than that reported to sterile cutaneous wounds. At 3 hpi, appreciable neutrophils are present, and their numbers increase across our study, suggesting the plateau phase may not be reached during the time scale of the experiment.

### Conclusions

Our gene-expression analysis reveals modulation of some pro-inflammatory genes at 1 and 3 hpi that intensifies to include genes related to antimicrobial responses, regulation of reactive oxygen species, wound healing, and signaling through AP-1, NFκB, MAPK, and G-protein pathways at 6 hpi. At 12 hpi, chemokines and cytokines consistent with a neutrophil-dominated immune response are prominent. Histopathology showed mast cell degranulation by 1 hpi and increasing neutrophil influx beginning at 3 hpi, supporting the array data. These results suggest early cutaneous host responses to tick feeding are more pro-inflammatory than expected, and highlight the importance of neutrophils and related pathways in tick engorgement and tick-borne viral transmission.

## Supporting Information

Figure S1 Histopathology of *Ixodes scapularis* nymphal bite sites at 1 hr PI. Skin biopsies were fixed in formaldehyde prior to decalcification and paraffin embedding. Sections were stained with Geimsa, as described in methods section. Mast cells appear as collections of dark purple granules.
(TIF)

Table S1 Correlation between the microarray and the real-time PCR analysis. FC, fold change; P val, p-value; hpi, hours post-infestation.
(DOCX)
