# Supplementary figures and images for: Early Immunologic Events at the Tick-Host Interface
Source: PLoS One. 2012 Oct 15;7(10):e47301. doi: 10.1371/journal.pone.0047301 (PMC3471850; doi:10.1371/journal.pone.0047301)

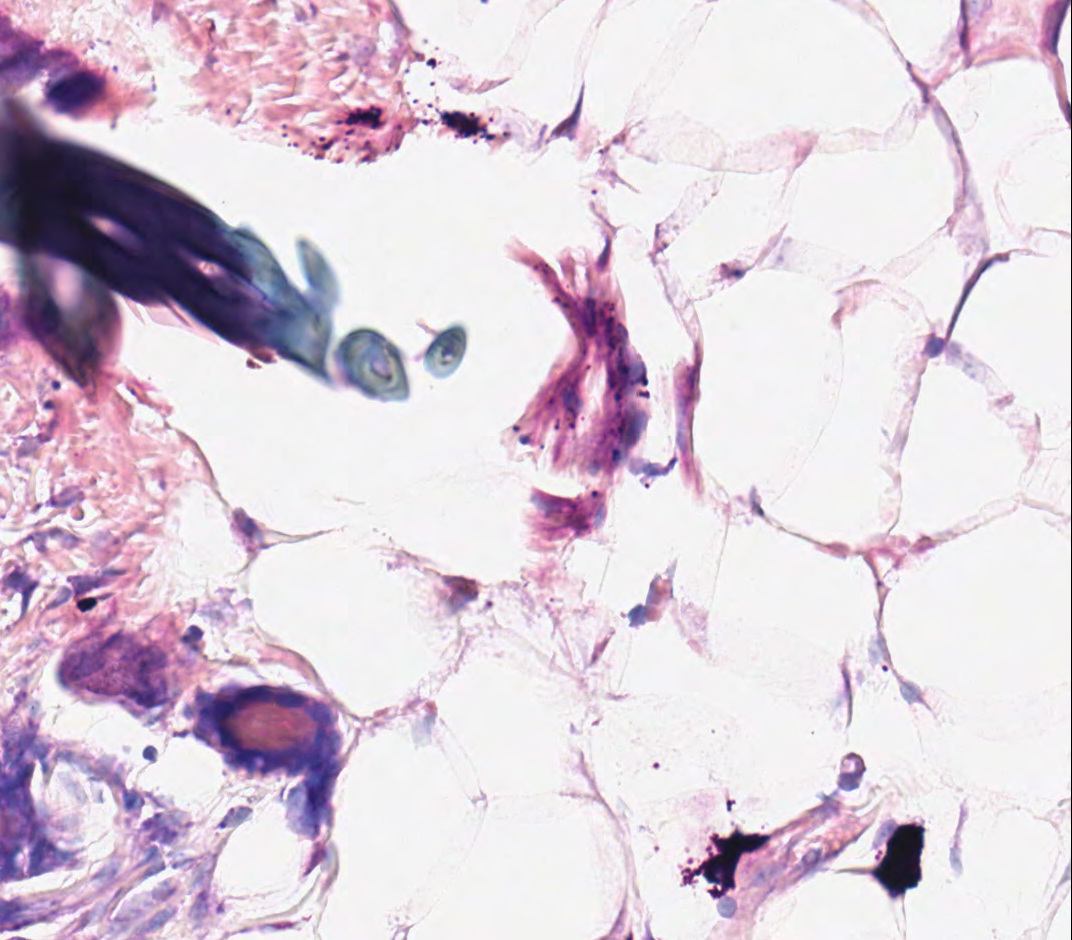

Supplement: Figure S1 — Histopathology of Ixodes scapularis nymphal bite sites at 1 hr PI. Skin biopsies were fixed in formaldehyde prior to decalcification and paraffin embedding. Sections were stained with Geimsa, as described in methods section. Mast cells appear as collections of dark purple granules. (TIF) [file pone.0047301.s001.tif]
