# Supplementary material for: Early Immunologic Events at the Tick-Host Interface
Source: PLoS One. 2012 Oct 15;7(10):e47301. doi: 10.1371/journal.pone.0047301 (PMC3471850; doi:10.1371/journal.pone.0047301)
Supplement: Table S1 — Correlation between the microarray and the real-time PCR analysis. FC, fold change; P val, p-value; hpi, hours post-infestation. (DOCX) [file pone.0047301.s002.docx]

|  | 1hpi array | | 1hpi validation | | 3hpi array | | 3hpi validation | | 6hpi array | | 6hpi validation | | 12hpi array | | 12hpi validation | |
| --- | --- | --- | --- | --- | --- | --- | --- | --- | --- | --- | --- | --- | --- | --- | --- | --- |
| Gene | FC | P val | FC | P val | FC | P val | FC | P val | FC | P val | FC | P val | FC | P val | FC | P val |
| Banf1 | -1.05 | 0.582 | 1.17 | 0.0455 | -1.13 | 0.195 | 1.03 | 0.657 | -1.07 | 0.46 | 1.02 | 0.812 | 1.53 | 2.09E-4 | 1.14 | 0.0934 |
| C1qb | -1.12 | 0.607 | 1.15 | 0.476 | 1.06 | 0.782 | 1.12 | 0.562 | 1.01 | 0.96 | 2.58 | 5.87E-6 | 1.46 | 0.0869 | 2.34 | 3.64E-5 |
| Ccl2 | -1.16 | 0.678 | 1.31 | 0.279 | -1.2 | 0.609 | 2.64 | 2.25E-4 | 1.39 | 0.365 | 6.58 | 2.71E-10 | 3.75 | 0.00124 | 8.76 | 3.34E-12 |
| Ccl7 | -1.34 | 0.352 | 1.20 | 0.411 | -1.18 | 0.605 | 2.78 | 3.02E-5 | 1.42 | 0.274 | 5.75 | 1.68E-10 | 4.02 | 2.26E-4 | 7.28 | 3.16E-12 |
| Ccr5 | -1.14 | 0.743 | 1.99 | 0.0259 | 1.11 | 0.8 | 2.84 | 9.67E-4 | 1.09 | 0.825 | 8.57 | 1.75E-09 | 2.42 | 0.0399 | 12 | 2.33E-11 |
| Clec4e | 1.07 | 0.769 | 1.39 | 0.516 | 1.11 | 0.644 | 1.99 | 0.177 | 1.46 | 0.115 | 3.09 | 0.0285 | 2.23 | 0.00243 | 6.44 | 4.72E-4 |
| Ctse | -1.12 | 0.765 | 1.16 | 0.727 | -1.07 | 0.858 | 1.46 | 0.366 | 1.24 | 0.58 | 1.84 | 0.148 | 4.11 | 0.0016 | 1.47 | 0.36 |
| Cxcl5 | 1.11 | 0.792 | -1.20 | 0.664 | 1.07 | 0.867 | 3.59 | 0.00412 | 1.25 | 0.591 | 50.2 | 9.12E-13 | 4.47 | 0.00133 | 96.4 | 3.44E-15 |
| Fn1 | 1.05 | 0.8 | 1.69 | 0.00668 | -1.28 | 0.252 | 1.72 | 0.00538 | -1.61 | 0.0328 | 2.12 | 1.75E-4 | -1.52 | 0.0573 | 1.66 | 0.00873 |
| Foxp1 | 1.43 | 2.55E-4 | -1.08 | 0.754 | 1.41 | 3.83E-4 | -1.01 | 0.966 | 1.25 | 0.0108 | -1 | 0.99 | 1.22 | 0.0215 | -1.13 | 0.587 |
| Ifng | -1.03 | 0.73 | 2.76 | 0.242 | -1.01 | 0.872 | 4.34 | 0.0926 | 1.03 | 0.724 | 4.16 | 0.102 | -1 | 0.964 | 2.83 | 0.231 |
| Il1b | 1.12 | 0.766 | 1.54 | 0.141 | 1.06 | 0.88 | 5.57 | 1.74E-7 | 1.97 | 0.0871 | 9.45 | 1.61E-10 | 3.85 | 0.00185 | 12.8 | 2.9E-12 |
| Il10 | 1.05 | 0.827 | 2.31 | 0.0186 | 1 | 0.985 | 2.51 | 0.00983 | 1.07 | 0.757 | 4.78 | 3.04E-5 | 1.13 | 0.589 | 3.12 | 0.00168 |
| Il3 | -1.09 | 0.426 | -2.50 | 0.246 | 1.03 | 0.789 | -2.08 | 0.352 | -1.03 | 0.8 | -1.31 | 0.73 | -1.02 | 0.84 | -2.27 | 0.3 |
| Il4 | -1.27 | 0.307 | -2.23 | 7.99E-4 | -1.08 | 0.737 | -1.33 | 0.216 | -1.09 | 0.701 | -1.3 | 0.255 | -1.06 | 0.791 | -1.39 | 0.152 |
| Il6 | 1.22 | 0.712 | -1.03 | 0.605 | -1.06 | 0.908 | 1.03 | 0.547 | 1.36 | 0.564 | -1.15 | 0.00717 | 7.13 | 0.00123 | -1.01 | 0.789 |
| Il2 | 1.01 | 0.879 | 1.09 | 0.758 | -1.03 | 0.592 | 2.08 | 0.0149 | 1.01 | 0.84 | 1.31 | 0.352 | -1.1 | 0.0469 | 1.01 | 0.968 |
| Il4ra | -1.01 | 0.972 | 2.45 | 3.51E-4 | 1.09 | 0.676 | 1.52 | 0.0792 | 1.12 | 0.603 | 3.63 | 1.04E-6 | 1.16 | 0.491 | 3.41 | 2.77E-6 |
| Jak1 | -1.05 | 0.538 | 1.06 | 0.475 | -1.07 | 0.367 | -1.02 | 0.839 | -1.2 | 0.0274 | -1 | 0.988 | -1.08 | 0.317 | -1.07 | 0.394 |
| Muc1 | -1.02 | 0.899 | 1.30 | 0.51 | 1.11 | 0.498 | 1.81 | 0.134 | 2.18 | 6.38E-5 | 1.52 | 0.287 | 1.01 | 0.947 | 1.88 | 0.113 |
| Myb | -1.03 | 0.856 | -1.38 | 0.0347 | 1.09 | 0.546 | 1.07 | 0.635 | 1.37 | 0.0424 | 1.04 | 0.767 | 1.94 | 1.8E-4 | -1.11 | 0.497 |
| Saa1 | 1.05 | 0.871 | 1.54 | 0.167 | 1.04 | 0.91 | 3.08 | 6.02E-4 | 1.46 | 0.222 | 4.97 | 3.06E-6 | 1.55 | 0.162 | 3.11 | 5.43E-4 |
| Sele | 1.03 | 0.847 | 1.5 | 0.0613 | -1.04 | 0.817 | 2.1 | 9.02E-4 | 1.24 | 0.185 | 3.78 | 5.17E-08 | 1.07 | 0.645 | 3.04 | 2.42E-6 |
| Serpina3n | -1.03 | 0.863 | 2.24 | 1.86E-5 | 1.29 | 0.179 | 2.89 | 8.59E-08 | 1.7 | 0.00936 | 3.97 | 7.7E-11 | 1.77 | 0.00565 | 3.86 | 1.41E-10 |
| Socs1 | -1.08 | 0.57 | -1.54 | 0.0387 | 1.01 | 0.915 | -1.96 | 0.00171 | 1.02 | 0.855 | -1.13 | 0.544 | 1.07 | 0.596 | -1.28 | 0.239 |
| Stat6 | 1.02 | 0.73 | -1.03 | 0.903 | -1.12 | 0.0483 | -1.11 | 0.696 | -1.08 | 0.163 | 1.04 | 0.874 | -1.15 | 0.0154 | -1.1 | 0.725 |
| Vapb | -1.15 | 0.0595 | 1.34 | 0.0887 | -1.05 | 0.519 | 1.28 | 0.153 | -1.18 | 0.0292 | 1.58 | 0.00945 | -1.07 | 0.338 | 1.33 | 0.102 |
| Vwf | -1.16 | 0.441 | 1.06 | 0.794 | -1.27 | 0.212 | 1.29 | 0.272 | -1.58 | 0.0209 | 1.23 | 0.381 | -1.67 | 0.0113 | -1.13 | 0.605 |
